# The Hype and Hope of Stem Cell Rejuvenation

**DOI:** 10.1093/geroni/igaa057.2983

**Published:** 2020-12-16

**Authors:** Melissa Harris

**Affiliations:** Unversity of Alabama, Birmingham, Birmingham, Alabama, United States

## Abstract

Adult stems play a major role in keeping our bodies in good working order by replenishing tissue as it wears out, and by providing new cells in the event of injury or damage. Unfortunately, the biological process of aging does not discriminate, and all cells, including our stem cells fall prey to getting older. Failing stem cells contribute to characteristics of aging and degenerative disease, and regenerative medicine research has focused on identifying ways in which stem cells can be used or rejuvenated to alleviate the symptoms of old age. Stem cell rejuvenation, however, has also inspired companies to provide services for harvesting and storing young stem cells with little real idea of how these stem cells might be used or their efficacy. In this seminar we will review what is known about the way in which stem cells fail, and the hype and hope surrounding stem cell rejuvenation.

